# Luciferase Reporter Gene Assay on Human, Murine and Rat Histamine H_4_ Receptor Orthologs: Correlations and Discrepancies between Distal and Proximal Readouts

**DOI:** 10.1371/journal.pone.0073961

**Published:** 2013-09-02

**Authors:** Uwe Nordemann, David Wifling, David Schnell, Günther Bernhardt, Holger Stark, Roland Seifert, Armin Buschauer

**Affiliations:** 1 Institute of Pharmacy, University of Regensburg, Regensburg, Germany; 2 Institute of Pharmaceutical and Medicinal Chemistry, Heinrich-Heine-Universität Düsseldorf, Düsseldorf, Germany; 3 Institute of Pharmacology, Medical School of Hannover, Hannover, Germany; University of South Florida College of Medicine, United States of America

## Abstract

The investigation of the (patho)physiological role of the histamine H_4_ receptor (H_4_R) and its validation as a possible drug target in translational animal models are compromised by distinct species-dependent discrepancies regarding potencies and receptor subtype selectivities of the pharmacological tools. Such differences were extremely pronounced in case of proximal readouts, e. g. [^32^P]GTPase or [^35^S]GTPγS binding assays. To improve the predictability of in vitro investigations, the aim of this study was to establish a reporter gene assay for human, murine and rat H_4_Rs, using bioluminescence as a more distal readout. For this purpose a cAMP responsive element (CRE) controlled luciferase reporter gene assay was established in HEK293T cells, stably expressing the human (h), the mouse (m) or the rat (r) H_4_R. The potencies and efficacies of 23 selected ligands (agonists, inverse agonists and antagonists) were determined and compared with the results obtained from proximal readouts. The potencies of the examined ligands at the human H_4_R were consistent with reported data from [^32^P]GTPase or [^35^S]GTPγS binding assays, despite a tendency toward increased intrinsic efficacies of partial agonists. The differences in potencies of individual agonists at the three H_4_R orthologs were generally less pronounced compared to more proximal readouts. In conclusion, the established reporter gene assay is highly sensitive and reliable. Regarding discrepancies compared to data from functional assays such as [^32^P]GTPase and [^35^S]GTPγS binding, the readout may reflect multifactorial causes downstream from G-protein activation, e.g. activation/amplification of or cross-talk between different signaling pathways.

## Introduction

The histamine H_4_ receptor (H_4_R) [1]–[5] is preferably expressed on cells of hematopoietic origin such as eosinophils and mast cells and supposed to be involved in inflammatory diseases, e.g. asthma, and pruritis [6]–[10]. To investigate the (patho)physiological role of the H_4_R translational, animal models for allergic asthma and allergic contact dermatitis in mice [11]–[15] or rat models for acute inflammation and conjunctivitis [16], [17] were used. Most of the studies confirmed the pro-inflammatory role of the H_4_R by blocking the H_4_R-mediated response with JNJ 7777120 (1-[(5-chloro-1H-indol-2-yl)carbonyl]-4-methylpiperazine), which is reported to be equipotent as an antagonist at the human, mouse and rat H_4_R orthologs [18].

However, there are also controversial reports. The administration of the H_4_R agonist 5(4)-methylhistamine was benefical in a murine asthma model [12], and JNJ 7777120 increased the ocular histamine concentration in a rat conjunctivitis model [17] (for a recent review cf. Neumann et al. [19]). Furthermore, the overall amino acid identities of H_4_R species orthologs are remarkably low (human versus mouse and rat: ∼70%) compared to other histamine receptor subtypes (H_1_R, H_2_R and H_3_R) [20]. Although relatively small differences in the sequence of histamine receptor species orthologs can result in different potencies and efficacies of individual ligands, the discrepancies are exceptionally high in case of the H_4_R [21]. In various in vitro assay systems the recombinantly expressed mouse and rat H_4_R revealed substantial species-dependent differences compared to the human receptor concerning affinity, potency and quality of action of pharmacological tools, compromising the predictive value with respect to translational animal models [20]–[23]. For example, in comparison to the human H_4_R, UR-PI294 (N^1^-[3-(1H-imidazol-4-yl)propyl]-N^2^-propionylguanidine) and UR-PI376 (2-cyano-1-[4-(1H-imidazol-4-yl)butyl]-3-[(2-phenylthio)ethyl]guanidine) [24], [25] displayed considerably lower potencies and efficacies (UR-PI376) in the [^32^P]GTPase and [^35^S]GTPγS binding assays on membrane preparations of Sf9 insect cells expressing the mouse or rat H_4_R [23]. Most strikingly, JNJ 7777120 exhibited stimulatory effects at the mouse and rat H_4_R in functional assays on Sf9 cell membranes [23]. Moreover, the use of JNJ 7777120 as standard antagonist in animal models was questioned due to stimulation of G-protein independent β-arrestin recruitment [26]. Biased signaling of the hH_4_R has also been shown for other H_4_R ligands [27].

**Figure 1:**
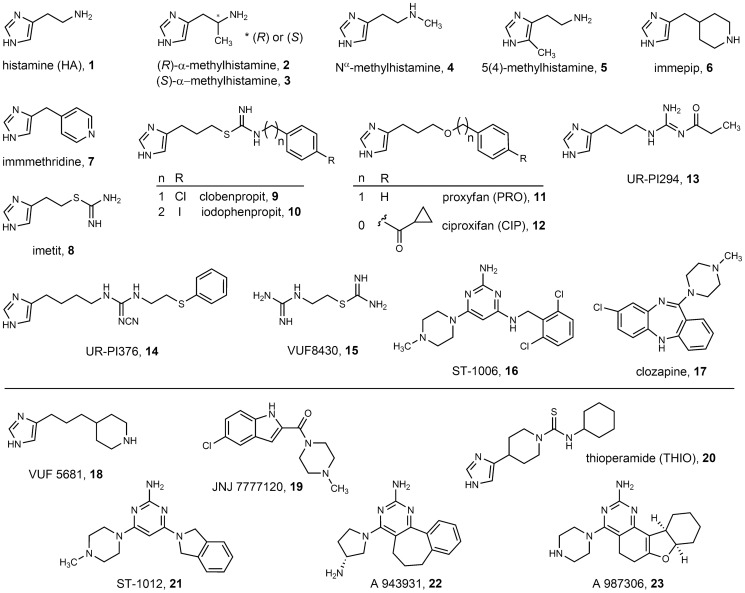
Chemical structures of the examined H_4_R ligands. Agonists (**1**–**17**), antagonists/inverse agonists (**18–23**) at the human H_4_R.

The aforementioned controversial findings underline the necessity to evaluate pharmacological tools at the H_4_R species orthologs of interest using different assay systems. For this purpose, a cAMP response element (CRE) controlled luciferase reporter gene assay in HEK293T cells, stably expressing the human, the mouse or the rat H_4_R, was established. The H_4_R is Gα_i/o_-coupled and reduces forskolin stimulated cyclic adenosine monophosphate (cAMP) formation after agonist binding [2]. The optimal concentration of forskolin used for pre-stimulation depends on the cell type [28] and should correspond to the EC_50_ of forskolin in the assay system [29]. Therefore, the potency of forskolin was determined, and the effect of the phosphodiesterase (PDE) inhibitor isobutylmethylxanthine (IBMX) was evaluated to optimize the sensitivity of the procedure. Due to the delayed onset of gene expression, incubation periods of four to six hours are required [30], increasing the risk of agonist mediated receptor desensitization, which can lead to a decrease in agonist potencies [30]. Therefore, the time course of the luciferase expression was determined to find the minimum incubation period required for appropriate signal strength. For validation, potencies and efficacies of 23 selected H_4_R ligands, comprising agonists, inverse agonists and antagonists, were determined (Figure 1).

## Materials and Methods

### Ethics Statement

Human embryonal kidney (HEK293T) cells were purchased from the German Collection of Microorganism and Cell Cultures (DSMZ, Braunschweig, Germany).

### Histamine Receptor Ligands

Histamine (HA, **1**) was purchased from Alfa Aesar (Karlsruhe, Germany). (R)-α-methylhistamine (**2**), (S)-α-methylhistamine (**3**), N^α^-methylhistamine (**4**), 5(4)-methylhistamine (**5**), immepip (**6**), immethridine (**7**), imetit (**8**), clobenpropit (**9**), iodophenpropit (**10**), proxyfan (PRO, **11**), ciproxifan (CIP, **12**), clozapine (**17**), VUF 5681 (**18**), A 943931 (**22**) and A 987306 (**23**) were from Tocris Bioscience (Ellisville, MO, USA), UR-PI294 (**13**), UR-PI376 (**14**), VUF 8430 (**15**), ST-1006 (**16**), JNJ 7777120 (**19**), thioperamide (THIO, **20**), and ST-1012 (**21**) were synthesized in our laboratories. Chemical structures of the ligands are depicted in Figure 1. Except for **14**, **16**, **17**, **21** and **23** all stock solutions (10 mM) were prepared in Millipore water. Stock solution of **17** and **23** were made in 20 mM HCl, whereas **14**, **16** and **21** were dissolved in 50% (v/v) dimethyl sulfoxide (DMSO). Stock solutions of **17** and **23** and those ligands dissolved in water were diluted with Dulbecco’s modified Eagle’s medium (DMEM) supplemented with 10% (v/v) fetal calf serum (FCS). The stock solutions of **14**, **16** and **21** were diluted with DMEM supplemented with 10% (v/v) FCS and 10% (v/v) DMSO.

### Subcloning of FLAG Epitope- and Hexahistidine-tagged mH_4_R cDNA into the Shuttle Vector pcDNA3.1(+)

The FLAG epitope (F)- and the hexahistidine (His_6_)-tagged mH_4_R cDNA cloned in pGEM-3Z [23] was subcloned at *Hind*III and *Xba*I restriction sites into pcDNA3.1(+), encoding G418 resistance. Double digestion with *Hind*III (Fermentas GmbH, St. Leon-Rot, Germany) and *Xba*I (Fermentas) restriction enzymes was performed in reaction buffer Tango (Fermentas) with a two-fold excess of *Hind*III at 37°C for 3 h. The DNA bands of the SF-mH_4_R-His_6_ (1336 bp) (S stands for the cleavable signal peptide from influenza hemagglutinin, F for flag) insert as well as the linearized pcDNA3.1(+) vector (5352 bp) were extracted from the 1% (m/v) agarose (pegGOLD Universal-Agarose, Peqlab, Erlangen, Germany) gel using the QIAquick Gel Extraction Kit (QIAGEN, Hilden, Germany) according to the manufacturer’s protocol. The ligation was performed using T4-DNA-Ligase (6 Weiss U/µL) (New England Biolabs, Ipswich, MA, USA). After the transformation of the ligation product (pcDNA3.1(+)SF-mH_4_R-His_6_) into competent *E. coli* (Top10 strain) cells and plating on agar (Roth, Karlsruhe, Germany) plates containing 100 µg/mL of ampicillin (Sigma, Munich, Germany), one resistant colony was chosen for large scale plasmid DNA preparation using the Qiagen Plasmid Purification kit (Qiagen, Hilden, Germany) according to the manufacturer’s instructions. The restriction analysis with *Hind*III and *Xba*I as well as the sequencing of the amplified pcDNA3.1(+)SF-mH_4_R-His_6_ vector (performed by Entelechon, Bad Abbach, Germany) confirmed the correct composition of the vector.

### Cell Culture and Transfection

HEK293T cells were cultured in Dulbecco’s Modified Eagle Medium (DMEM) (Sigma) containing L-glutamine, 4500 mg/L glucose, 3.7 g/L NaHCO_3_ (Merck, Darmstadt, Germany), 110 mg/L sodium pyruvate (Serva, Heidelberg, Germany) and 10% (v/v) fetal calf serum (FCS) (Biochrom, Berlin, Germany). The HEK293T cells, stably expressing the tagged human H_4_ receptor (HEK293T-SF-hH_4_R-His_6_), were cultured in the above-mentioned medium supplemented with 600 µg/mL geneticin (G418) (Biochrom). Cells were maintained at 37°C and 5% CO_2_ in a water-saturated atmosphere in 75-cm^2^ culture flasks (Sarstedt, Nümbrecht, Germany) and diluted (1∶10) twice a week with fresh medium.

HEK293T-SF-hH_4_R-His_6_ cells were stably co-transfected with pGL4.29[luc2P/CRE/Hygro] (Promega, Mannheim, Germany) encoding hygromycin resistance (Hygro) and the firefly luciferase (luc2P), the transcription of which is controlled by the cAMP responsive element (HEK293T-SF-hH_4_R-His_6_-CRE-Luc cells). HEK293T cells were stably co-transfected with pGL4.29[luc2P/CRE/Hygro] (HEK293T-CRE-Luc cells) and pcDNA3.1(+)SF-mH_4_R-His_6_ (HEK293T-SF-mH_4_R-His_6_-CRE-Luc) or pcDNA3.1(+)-SF-rH_4_R-His_6_ (HEK293T-SF-rH_4_R-His_6_-CRE-Luc cells), respectively. For transfection, the cells were seeded into a 24 well-plate (Becton Dickinson, Heidelberg, Germany), so that they reached 60–70% confluenccy on the next day. The transfection mixture containing 0.5 µg of the DNA and either 1 µL (4∶2 ratio), 1.5 µL (6∶2 ratio) or 2 µL (8∶2 ratio) of FuGene®HD transfection reagent (Roche Diagnostics, Mannheim, Germany) was prepared according to the manufacturer’s protocol and added to the cells, followed by an incubation period of 36–48 h at 37°C and 5% CO_2_ in a water-saturated atmosphere. Co-transfected cells were cultured in DMEM supplemented with 10% (v/v) FCS, 600 µg/mL of G418 and 200 µg/mL of hygromycin B (A.G. Scientific, San Diego, USA).

### Luciferase Reporter Gene Assay

Approximately 2 · 10^5^ transfected cells, suspended in DMEM supplemented with 10% (v/v) FCS, were seeded per well into flat-bottomed 96-well plates (Greiner, Frickenhausen, Germany). The cells were allowed to attach for 17 h at 37°C, 5% CO_2_ in a water-saturated atmosphere. A stock solution (10 mM) of forskolin (Sigma) in DMSO was used to prepare feed solutions in DMEM containing 10% (v/v) FCS (final DMSO concentration in the assay was ≤1%). For experiments in the presence of a PDE inhibitor, the feed solution of forskolin contained 500 µM of IBMX (Sigma).

After addition of forskolin (0.4 µM for the cells expressing the human H_4_R and 1 µM for the rat and mouse H_4_R expressing cells) alone (to determine forskolin potency) or in combination with histaminergic ligands, the cells were incubated for 5 h. In antagonist mode, the forskolin solution was supplemented with 0.10, 0.15 or 1.00 µM of histamine as the agonist for the human, mouse and rat H_4_R expressing cells, respectively. Thereafter, the medium was discarded, the cells were washed once with 100 µL of phosphate buffered saline (PBS, pH 7.4) (KCl 2.7 mM; KH_2_PO_4_ 1.5 mM; NaCl 137 mM; Na_2_HPO_4_ 5.6 mM; NaH_2_PO_4_ 1.1 mM in Millipore water; all chemicals were from Merck, Darmstadt, Germany) and lysed in 40 µL of lysis buffer (pH 7.8) (N-[2-hydroxy-1,1-bis(hydroxymethyl)ethyl]glycine (Tricine) 25 mM (Sigma); glycerol 10% (v/v) (Merck); ethyleneglycoltetraacetic acid (EGTA) 2 mM (Sigma); Triton™ X-100 1% (v/v) (Serva); MgSO_4_ · 7H_2_O, 5 mM (Merck); dithiotreitol (DTT) 1 mM (Sigma)) for 45–60 min under shaking (180 rpm). For luminescence measurement, 20 µL of lysate were transferred into a white flat-bottomed 96-well plate (Greiner) and the GENios Pro microplate reader (Tecan, Salzburg, Austria) was primed with the luciferase assay buffer (pH 7.8) (glycyl-glycine (Gly-Gly) 25 mM; MgSO_4_ · 7H_2_O, 15 mM; KH_2_PO_4_, 15 mM (Merck); EGTA, 4 mM; adenosine 5′-triphosphate (ATP) disodium salt, 2 mM (Sigma); DTT 2 mM; D-luciferin potassium salt 0.2 mg/mL (Synchem, Felsberg, Germany)) [31]. Light emission was induced by the injection of 80 µL of the luciferase assay buffer into each well. Luminescence, expressed as RLUs (relative light units), was measured for 10 s. The basal luciferase activity was subtracted from each signal. EC_50_ and IC_50_ values were analyzed by nonlinear regression and best fitted to sigmoidal concentration-response curves with GraphPad Prism 5.04 (Graph Pad, San Diego (CA), USA). IC_50_ values were converted to K_B_ values using the Cheng-Prussoff equation [32]. The intrinsic activity of ligands was referred to the maximal response to histamine (HA), defined as α = 1 (full agonist). Agonist potencies are given as pEC_50_ values and antagonist activities were calculated as pK_B_ values. Measured RLUs were converted to percentual values referred to the span between the maximum effect induced by forskolin and the residual luciferase activity in the presence of histamine at the highest tested concentration. All data are means ± SEM of N independent experiments, each performed in triplicate. For monitoring the time course of the luciferase expression, transcription was stimulated with 10 µM of forskolin, and the cells were lysed after various incubation periods. For analysis, the respective basal RLUs were subtracted from each value and plotted against the time. For Schild analysis, concentration ratios (r) were obtained by dividing the EC_50_ concentrations of agonist in the presence of JNJ 7777120 (antagonist) by the EC_50_ concentration of agonist in the absence of JNJ 777120. The log (r - 1) values were plotted against the corresponding log [JNJ 7777120] values according to the Schild equation [33] and analyzed by linear regression with GraphPad Prism 5.04. The pA_2_ values were obtained from the intercept of the Schild plot with the x-axis.

### [^35^S]GTPγS Binding Assay

Cell culture and generation of high-titer recombinant baculovirus stocks as well as the co-infection of Sf9 cells with high-titer baculovirus stocks encoding Gα_i2,_ Gβ_1_γ_2_ and the respective H_4_R were performed as described recently [34], [35]. Membrane preparations were performed according to Gether et al. (1995) [36] in the presence of 0.2 mM phenylmethylsulfonyl fluoride, 1 mM ethylenediaminetetraacetic acid (EDTA), 10 µg/mL leupeptin and 10 µg/mL benzamidine as protease inhibitors. Prepared membranes were resuspended in binding buffer (75 mM Tris/HCl, 12.5 mM MgCl_2_,1 mM EDTA, pH 7.4) and stored at −80°C in 0.5 or 1.0 mL aliquots.

Membranes were thawed, centrifuged for 10 min at 4°C and 13,000 g and carefully resuspended in binding buffer. Experiments were performed in 96-well plates in a total volume of 100 µL per well. Each tube contained 8–12 µg of protein, 1 µM GDP, 100 mM NaCl, 0.05% (*w/v*) bovine serum albumine (BSA), 20 nCi of [^35^S]GTPγS (≥0.2 nM) and ligand at various concentrations. Neutral antagonists were incubated in the presence of histamine at concentrations corresponding to the 10-fold of the EC_50_ value at the respective receptor. Nonspecific binding was determined in the presence of 10 µM unlabeled GTPγS. After incubation under shaking at 200 rpm at room temperature for 2 h, bound [^35^S]GTPγS was separated from free [^35^S]GTPγS by filtration through glass microfibre filters using a 96-well Brandel harvester (Brandel Inc., Unterföhring, Germany). The filters were washed three to four times with cold binding buffer (4°C), dried over night and impregnated with meltable scintillation wax prior to counting with a Micro Beta2 1450 scintillation counter (Perkin Elmer, Rodgau, Germany).

Ligands were tested in triplicate. The maximal response to histamine was set to 100% and all other ligands were referenced to histamine.

## Results

### Optimization of the Assay Conditions

**Figure 2:**
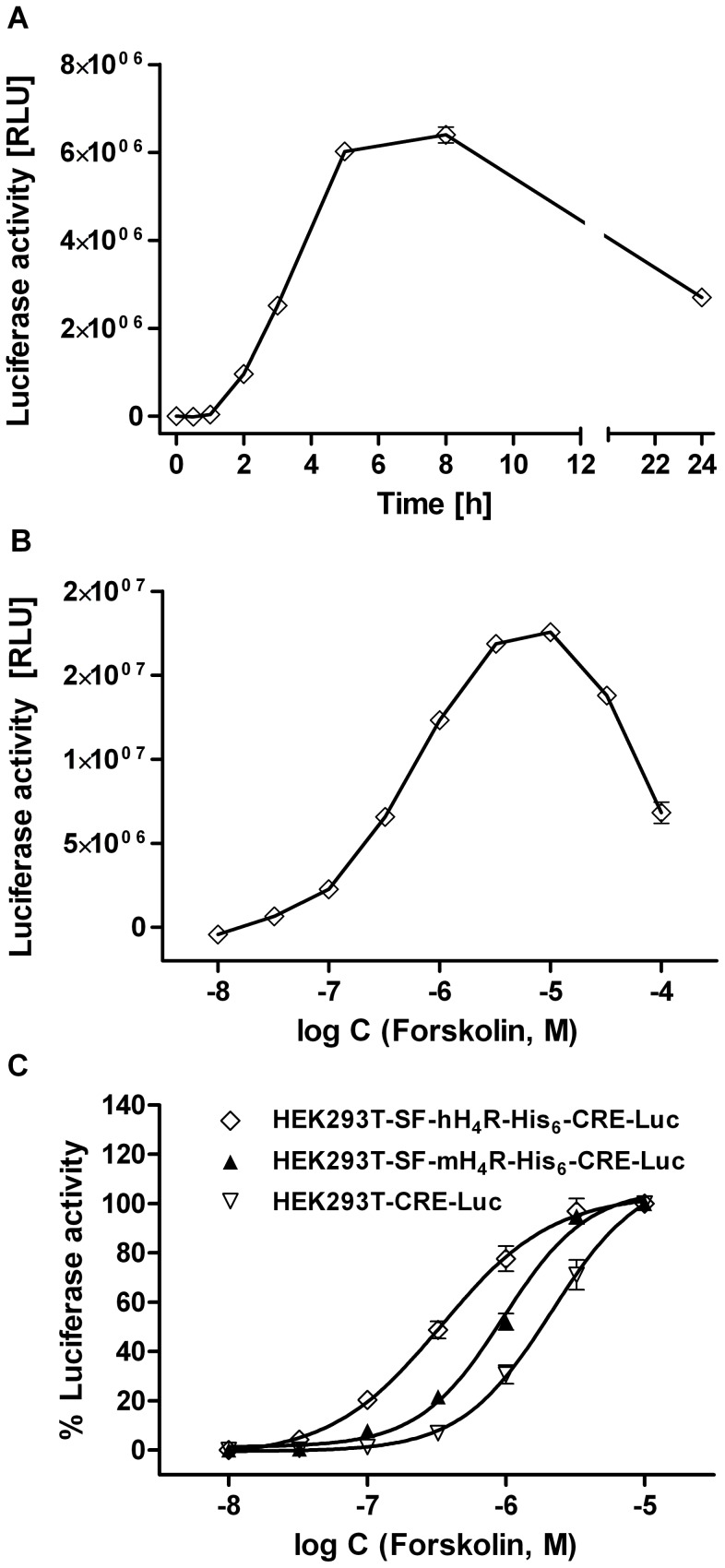
Stimulation of luciferase activity by forskolin. (A) Representative time course of the luciferase expression in HEK293T-CRE-Luc cells, stably expressing the CRE-controlled luciferase, upon stimulation with 10 µM of forskolin. The luciferase activity was determined after the indicated incubation periods (mean values ± SEM; n = 9). (B) Representative “bell-shaped” concentration-response curve obtained with HEK293T-SF-hH_4_R-His_6_-CRE-Luc cells, stably expressing the hH_4_R and the CRE-controlled luciferase. (C) Concentration response curves covering the ascending region of the signal obtained with different transfectants.

**Figure 3:**
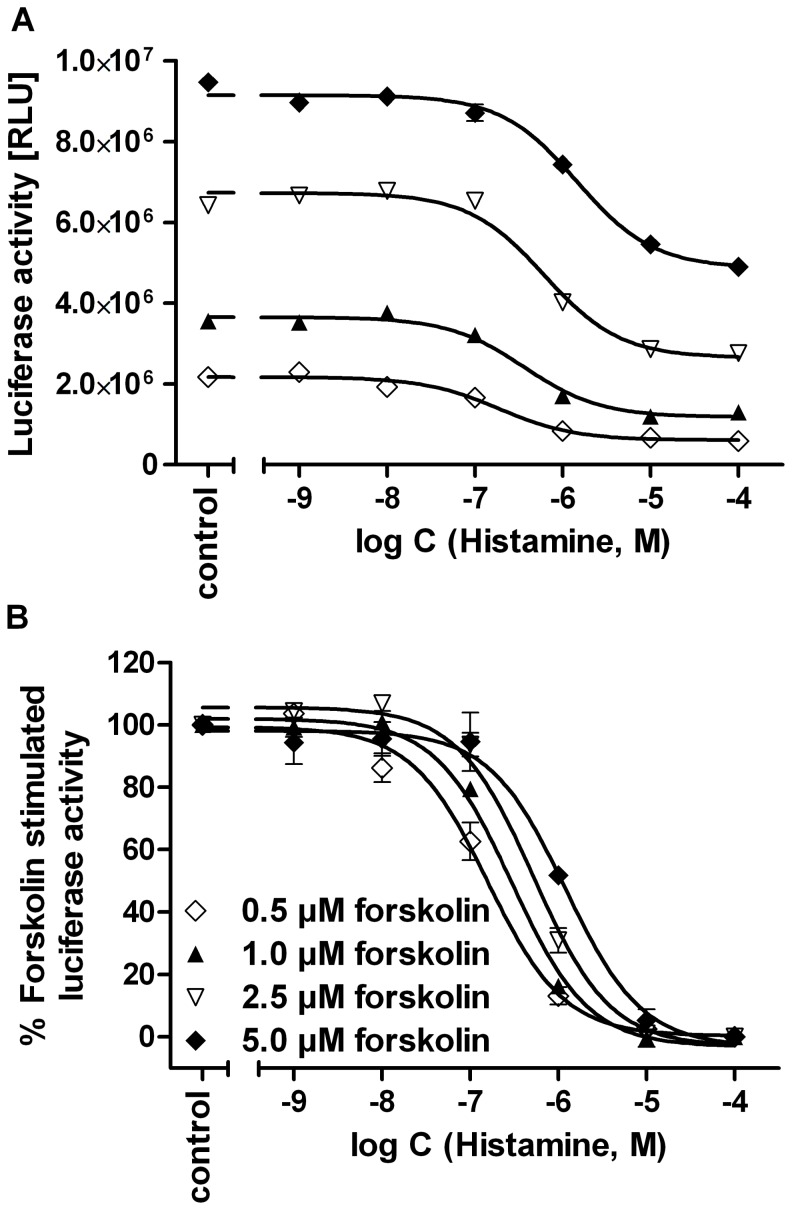
Inhibition of luciferase activity by histamine in rH_4_R expressing cells. Gα_i/o_ mediated inhibition of forskolin (0.5 µM–5.0 µM) stimulated luciferase activities by histamine (HA) in HEK293T-SF-rH_4_R-His_6_-CRE-Luc cells, stably expressing the rH_4_R and the CRE-controlled luciferase. (A) Representative luciferase reporter gene with RLU values as readout. (B) Normalized inhibition of forskolin stimulated luciferase activity (100%) by histamine (HA), with the maximum inhibitory effect of which set at 0%. Data points shown are the mean ± SEM of at least three independent experiments performed in triplicate.

In order to detect a Gα_i_-mediated inhibitory effect on the adenylyl cyclase (AC) activity, the reporter gene assay was performed in the presence of the AC stimulator forskolin. The time course of the luciferase expression upon stimulation with 10 µM forskolin is shown in Figure 2A. After a latency period of 0.5–1 h, the enzyme activity steeply increased, and a maximum was reached after 8 h. An incubation period of 5 h was sufficient to obtain 76–94% of the maximum expression. To optimize assay performance, the pEC_50_ value of forskolin in the respective cAMP reporter gene assay system [29] was determined (Figure 2). As the concentration-response curve shows an optimum (Figure 2B), only the ascending part of the curve was considered up to a forskolin concentration of 10 µM (Figure 2C). Interestingly, the potency of forskolin was significantly different: pEC_50_ values were 6.41±0.05 and 5.95±0.04 in the hH_4_R and mH_4_R co-transfected cells, respectively, and 5.50±0.11 in the HEK293T-CRE-Luc cells (Figure 2C). Forskolin concentration-dependently increased the luciferase expression in HEK293T-SF-rH_4_R-His_6_–CRE-Luc cells, which was inhibited by histamine (**1)** (Figure 3A) with pEC_50_ values of 6.81±0.11, 6.53±0.04, 6.29±0.07 and 5.91±0.04 (Figure 3B) at forskolin concentrations of 0.5, 1.0, 2.5 and 5 µM, respectively. Therefore, a concentration of 0.4 µM of forskolin was used for pre-stimulating the hH_4_R expressing cells, whereas 1 µM of forskolin was considered optimal for AC stimulation in mH_4_R and rH_4_R expressing cells. With respect to comparability of concentration-response curves of H_4_R ligands at H_4_R orthologs, the difference between maximum forskolin stimulation in the absence and the presence of the reference agonist histamine (100 µM) was set to 100% (Figure 3B).

**Figure 4:**
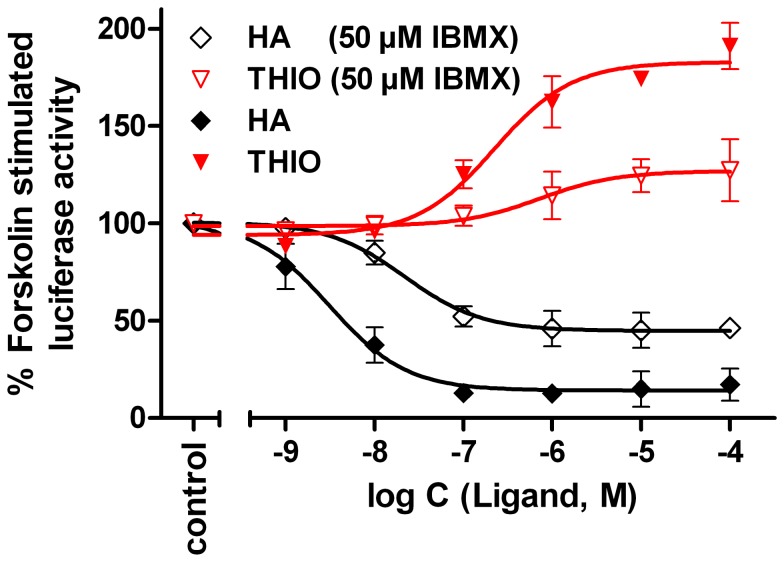
Effect of histamine and thioperamide on the luciferase activity in hH_4_R expressing cells. Concentration-response curves of histamine (HA) and thioperamide (THIO) on HEK293T-SF-hH_4_R-His_6_-CRE-Luc cells, stably co-expressing the CRE-controlled luciferase and the hH_4_R. The cells were pre-stimulated with 500 nM of forskolin alone or in combination with IBMX (50 µM). The effect of forskolin or that of forskolin plus IBMX was defined as 100% luciferase activity. Data points shown are the mean ± SEM of two independent experiments performed in triplicate.

In the presence of the PDE inhibitor IBMX (50 µM) the concentration-response curve of forskolin on HEK293T-SF-hH_4_R-His_6_-CRE-Luc-cells was shifted to the left, resulting in an pEC_50_ value of 6.86±0.06 (N = 3). Additionally, IBMX increased the receptor-independent luciferase activity by about a factor of four (data not shown). To investigate the effect of IBMX on the concentration-response curve of the full H_4_R agonist histamine (**1**) and the H_4_R inverse agonist thioperamide (**20**) HEK293T-hH_4_R-His_6_-CRE-Luc cells were pre-stimulated with forskolin (0.5 µM) alone or in combination with IBMX (50 µM) (cf. Figure 4). The maximum responses to histamine (**1**) and thioperamide (**20**), and thus the range of the signals, were reduced in the presence of IBMX. Therefore, further experiments were performed in the absence of IBMX.

### Functional Activity of H_4_R Ligands at the Human, Mouse and Rat H_4_R

A set of ligands (Figure 1), generally accepted as agonists (**1–17**), neutral antagonists or inverse agonists (**18–23**) at the human H_4_R was selected for functional investigations. The results from the reporter gene assays performed with the H_4_R species orthologs are summarized in Table 1 and compared to functional data from the [^35^S]GTPγS binding assay and the literature in Table 2.

**Table 1:** Potencies and efficacies of H_4_R ligands in the luciferase reporter gene assay at the hH_4_R, the mH_4_R and the rH_4_R.

	hH_4_R			mH_4_R			rH_4_R		
Ligand	pEC_50_ or (pK_B_)	α	N	pEC_50_ or (pK_B_)	α	N	pEC_50_ or (pK_B_)	α	N
Histamine (**1**)	7.77±0.12	1.00	6	7.06±0.13	1.00	4	6.53±0.04	1.00	6
(R)-α-Methylhistamine (**2**)	6.47±0.09	1.03±0.04	5	6.16±0.07	0.98±0.01	6	5.60±0.12	0.96±0.02	3
(S)-α-Methylhistamine (**3**)	5.22±0.09	0.90±0.04	5	4.72±0.16	0.82±0.07	3	4.26±0.04	0.69±0.03	3
N^α^-Methylhistamine (**4**)	6.74±0.12	0.98±0.03	4	6.24±0.13	0.97±0.02	3	6.23±0.09	0.98±0.04	3
5(4)-Methylhistamine (**5**)	7.25±0.05	0.97±0.03	3	6.87±0.05	0.97±0.02	4	6.03±0.05	1.00±0.03	3
Immepip (**6**)	7.64±0.12	0.98±0.02	5	6.85±0.17	0.95±0.03	3	7.17±0.06	0.93±0.05	3
Immethridine (**7**)	6.12±0.20	0.65±0.02	3	5.95±0.03	0.87±0.02	3	5.80±0.13	0.94±0.01	3
Imetit (**8**)	7.54±0.12	0.94±0.02	5	7.41±0.11	0.96±0.02	3	7.21±0.12	0.95±0.01	4
Clobenpropit (**9**)	7.87±0.07	0.97±0.03	3	6.73±0.08	0.55±0.05	3	6.80±0.11	0.37±0.03	3
Iodophenpropit (**10**)	7.30±0.14	0.73±0.02	4	(6.66±0.03)	0.01±0.05	3	(6.49±0.11)	−0.01±0.06	3
Proxyfan (**11**)	6.93±0.06	0.68±0.02	4	6.10±0.07	0.88±0.04	3	5.67±0.13	0.76±0.03	3
UR-PI294 (**13**)	8.74±0.11	0.98±0.02	6	8.29±0.18	0.97±0.02	5	8.16±0.03	1.03±0.02	3
UR-PI376 (**14**)	7.70±0.07	1.02±0.02	4	6.61±0.25	0.51±0.05	3	(5.15±0.05)	0.08±0.10	3
VUF 8430 (**15**)	7.04±0.10	0.97±0.04	3	6.83±0.03	0.96±0.02	3	6.06±0.06	0.98±0.02	3
ST-1006 (**16**)	8.05±0.05	0.91±0.01	3	7.76±0.11	0.37±0.04	4	6.08±0.17	−0.55±0.12	3
Clozapine (**17**)	6.96±0.14	1.30±0.05	8	5.44±0.06	0.99±0.01	3	5.70±0.11	1.12±0.05	4
VUF 5681 (1**8**)	(6.16±0.20	0.09±0.00	3	5.20±0.15	0.42±0.02	3	n.d.	–	–
JNJ 7777120 (**19**)	(7.81±0.19)	−0.31±0.06	3	(7.58±0.13)	−0.23±0.03	4	8.21±0.10	0.49±0.05	5
Thioperamide (**20**)	6.92±0.10	−0.32±0.04	6	6.52±0.13	−0.44±0.02	4	(6.89±0.14)	−0.20±0.02	4
ST-1012 (**21**)	7.26±0.05	−0.39±0.03	3	7.49±0.09	0.24±0.05	4	8.12±0.08	0.24±0.07	4
A 943931 (**22**)	7.58±0.12	−0.63±0.07	6	n.d.	–	–	(6.79±0.11)	−0.06±0.00	6
A 987306 (**23**)	7.17±0.07	−0.62±0.07	4	n.d.	–	–	(7.85±0.13)	−0.08±0.00	6

Data are represented as mean values ± SEM of N independent experiments performed in triplicate. α: intrinsic activity, referred to histamine = 1.00; n.d.: not determined.

**Table 2:** Reference data of H_4_R ligands determined in the [^35^S]GTPγS binding assay at the hH_4_R, the mH_4_R and the rH_4_R and reported in literature.

	hH_4_R		mH_4_R		rH_4_R	
Ligand	pEC_50_ or (pK_B_)	α	pEC_50_ or (pK_B_)	α	pEC_50_ or (pK_B_)	α
Histamine (**1**)	7.1–8.2a,e,j,l,m	1.0	5.2–7.5a,d,e,f, l	1.0	4.3–7.1a,d,e,f,l	1.0
(R)-α-Methylhistamine (**2**)	6.2–7.0e,j	0.8–1.0	6.6^e^	0.8	6.0^e^	0.4
(S)-α-Methylhistamine (**3**)	4.9^j^	1.0	–	–	––	–
N^α^-Methylhistamine (**4**)	6.1–7.4e,j	0.9–1.0	–	–	–	–
5(4)-Methylhistamine (**5**)	7.2–7.8^d,j,^ m	0.9–1.0	6.02^d^	1.0	5.1^d^	1.1
Immepip (**6**)	7.7–7.8a,j	0.8–0.9	5.27^a^	0.7	5.0^a^	0.7
Immethridine (**7**)	6.0^j^	0.5	–	–	–	–
Imetit (**8**)	7.9–8.5e,j	0.3–0.9	8.1^e^	0.8	8.1^e^	0.3
Clobenpropit (**9**)	7.7–8.3a,j,m	0.5–1.3	6.1^a^	0.2	(6.3)^a^	0.0
Iodophenpropit (**10**)	(7.7–8.0)a,j	0.0	(6.4)^a^	0.0	(6.0)^a^	0.0
Proxyfan (**11**)	7.2^j^	0.5	–	–	–	–
UR-PI294 (**13**)	8.4–8.5a,d	0.9–1.0	6.1–6.5a,d	1.0	4.6–5.5a,d	1.0–1.6
UR-PI376 (**14**)	7.5–7.8a,d,m	0.9–1.3	(6.1)^a^–6.9^d^	0.0–0.2	(5.5)^a^–4.5^d^	0.0–0.4
VUF 8430 (**15**)	7.3–8.2a,k,m	0.8–1.0	5.1^a^	0.7	4.5^a^	0.4
ST-1006 (**16**)	8.9^c^	0.2	–	–	–	–
Clozapine (**17**)	5.8–6.8a,b,j,m	0.7–1.2	<4^a^	0.0	<4^a^	0.0
VUF 5681 (1**8**)	<5^i^	–	–	–	–	–
JNJ 7777120 (**19**)	(7.6)^a^–7.5^d^	−0.4^d^	6.1–6.7a,d	0.4–0.6	6.1–6.5a,d	0.2–0.5
Thioperamide (**20**)	6.4–7.0a,d,j,m	–1.0 – −1.4a,d	(7.1)^a^	0.0	(6.4)^a^	0.0
ST-1012 (**21**)	7.4^c^	−1.1	–	–	–	–
A 943931 (**22**)	(8.2)^g^–7.3^a^	−1.8^a^	(8.2)^g^	0.0	(6.2–8.0)a,g	0.0
A 987306 (**23**)	(8.3)^h^–7.1^a^	−1.5^a^	(8.2)^h^	0.0	(7.1–8.3)a,h	0.0

Reference data are taken from (unless otherwise noted, α values referred to histamine = 1.0):

a functional [^35^S]GTPγS-binding assay on Sf9 cell membranes co-expressing the hH_4_R, mH_4_R or rH_4_R+G_iα2_+ β_1γ2_;

b,c,d Steady-state [^32^P]GTPase assay on Sf9 cell membranes co-expressing: hH_4_R-RGS19+ G_iα2_+ β_1γ2_^b^ [43], hH_4_R-GAIP+G_iα2_+ β_1γ2_, rH_4_R or mH_4_R+G_iα2_+ β_1γ2_+ GAIP ^d^ [23], hH_4_R+G_iα2_+ β_1γ2_^c^ (α value of ST-1012 referred to thioperamide = −1.0, [39]);

e calcium mobilization assay in 293-EBNA cells transiently co- expressing the hH_4_R, mH_4_R or rH_4_R with G_qi5_ [20];

f,g,h calcium mobilization assay in HEK293 cells stably co-expressing the hH_4_R, mH_4_R or rH_4_R with G_qi5_^f^ [46], ^g^ [44], ^h^ [45];

i,j,k,l CRE-β-galactosidase reporter gene assay in SK-N-MC cells stably co-expressing: the hH_4_R [40]–[42] or the mH_4_R ^k^ [57] with the CRE-β-galactosidase reporter gene;

m CRE-luciferase reporter gene assay in HEK293T cells, transiently co-expressing the hH_4_R with the CRE-controlled luciferase reporter gene [27];

l SRE-luciferase reporter gene assay in HEK293 cells, co-expressing the human, mouse or rat H_4_R+SRE-luciferase+Gα_qi_ chimeric G-protein [57].

#### hH_4_R agonists (compounds 1–17)

**Figure 5:**
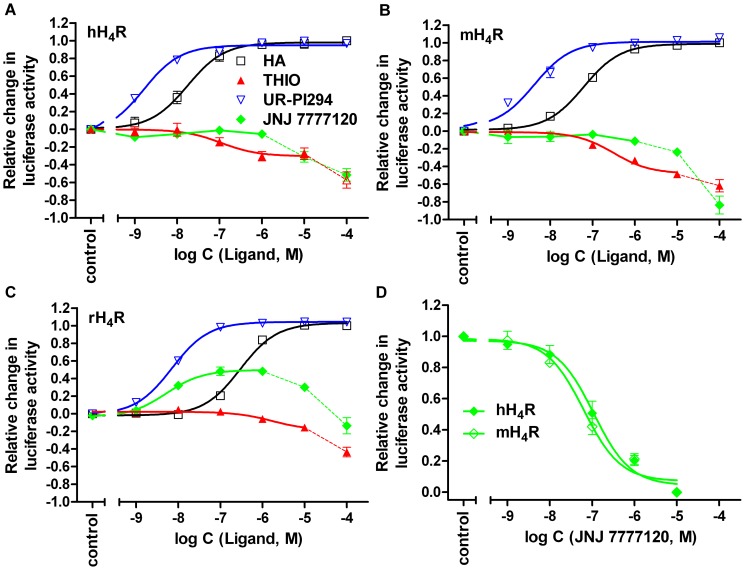
Effect of selected standard ligands on H_4_R orthologs. (A) Potencies and efficacies of histamine (HA), thioperamide (THIO), UR-PI294 and JNJ 7777120 at the hH_4_R, (B) the mH_4_R and (C) the rH_4_R (agonist mode). (D) Reversal of the HA (100–150 nM) mediated inhibition of the forskolin-stimulated luciferase activity by JNJ 7777120 at the hH_4_R and the mH_4_R (antagonist mode), in the luciferase reporter gene assay in HEK293T cells. Reaction mixtures contained ligands at the concentrations indicated on the abscissa to achieve saturated concentration response curves. Data points shown are the mean ± SEM of at least three independent experiments performed in triplicate. Data points connected by dashed lines reflect H_4_R-independent increase in luciferase activity at high ligand concentrations. The corresponding values were therefore excluded from non-linear correlations (D).

**Figure 6:**
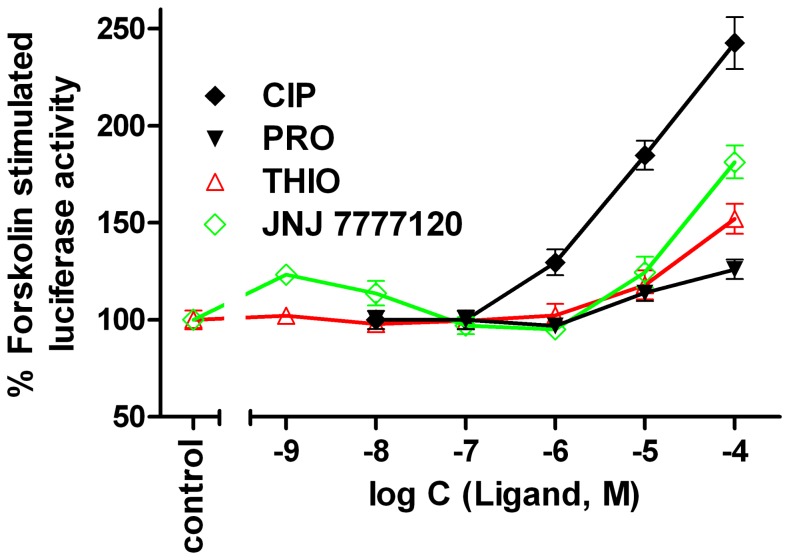
H_4_R-independent cellular effects of selected ligand. Representative H_4_R-independent increase in the forskolin (1 µM) stimulated luciferase activity by ciproxyfan (CIP), proxyfan (PRO), JNJ 7777120 and thioperamide (THIO) in HEK293T-CRE-Luc cells, stably expressing the CRE-controlled luciferase and devoid of the H_4_R.

The endogenous agonist histamine (**1**) inhibited forskolin stimulated luciferase activity with pEC_50_ values of 7.77, 7.06 and 6.53 in the hH_4_R, mH_4_R and rH_4_R expressing reporter cells, respectively (Table 1). The methyl-substituted analogs of histamine (**2**–**5**) acted, with the exception of **3**, as full agonists at the three H_4_R orthologs. Compared to the hH_4_R, a trend towards decreased potency was detected at the rodent receptors for compounds **1**–**5** (Figure 5A, B, C). Among the enantiomers **2** and **3**, (*R*)-α-methylhistamine (**2**) was the eutomer at all species orthologs. Compared to immepip (**6**), the pyridine analog immethridine (**7**) showed significantly reduced potency and intrinsic activity at the hH_4_R. By contrast, immethridine (**7**) exhibited almost full agonist activity at both, the mouse and rat H_4_R, with similar moderate potency compared to the hH_4_R. Imetit (**8**) exhibited almost the same potency and efficacy at the three H_4_R orthologs. In contrast, clobenpropit (**9)** and iodophenpropit (**10**), which can be considered as analogs of imetit (**8**) with an increased distance between the basic moieties and a large lipophilic group in the side chain, displayed a clear decrease in potency and maximal response at the mouse and rat H_4_R compared to the hH_4_R. Clobenpropit (**9**) was a potent full agonist at the hH_4_R and only a moderate partial agonist at the mouse and rat H_4_R, whereas iodophenpropit (**10**) acted as a partial agonist at the hH_4_R and a neutral antagonist at both, the mouse and the rat H_4_R. Proxyfan (**11**) partially activated the three H_4_R orthologs with significantly lower potencies on the rodent receptors. Whereas H_4_R-independent effects of **11** were negligible at concentrations >10 µM, the structural analog ciproxifan (**12**) induced a strong increase (by up to 250%) in luciferase activity at concentrations from 1 to 100 µM in HEK293-CRE-Luc cells devoid of H_4_R expression (Figure 6). Therefore, functional activities of **12** on H_4_R orthologs were not determined in the luciferase assay. The non-selective acylguanidine-type H_3/4_R agonist UR-PI294 (**13**) fully activated the human, mouse and rat H_4_R (Figure 5A, B, C), being the most potent agonist at all three H_4_R orthologs (Table 1). In contrast, the selective cyanoguanidine-type H_4_R agonist UR-PI376 (**14**) acted as a potent full hH_4_R agonist, exhibited only partial agonistic activity at the mH_4_R and was devoid of agonism at the rH_4_R (Table 1). VUF 8430 (**15**) had about the same potency at both, the mH_4_R and the hH_4_R, whereas the potency at the rH_4_R was distinctly lower. At all three H_4_R species orthologs, VUF8430 (**15**) was almost as efficacious as histamine (α = 0.96–0.98). The aminopyrimidine-type compound ST-1006 (**16**) exhibited pronounced differences in the quality of action at the H_4_R orthologs with nearly full agonism at the hH_4_R, partial agonism at the mH_4_R and inverse agonism at the rH_4_R. The antipsychotic drug clozapine (**17**) exhibited only moderate agonistic potency at the hH_4_R. However, with an α value of 1.30, clozapine was even more efficacious than histamine (**1**). Furthermore, clozapine (**17**) fully activated both, the mouse and the rat H_4_R, though with low pEC_50_ values (Table 1).

#### hH_4_R antagonists and inverse agonists (18–23)

Interestingly, VUF 5681 (**18**), with a spacer extended by two carbon atoms compared to the H_4_R agonist immepip (**6**), displayed no agonistic activity at the hH_4_R and only partial agonism at the mH_4_R. In the antagonist mode at the hH_4_R, VUF 5681 (**18**) inhibited the histamine-induced decrease in luciferase activity with a pK_B_ value of 6.16±0.20. JNJ 7777120 (**19**) behaved as neutral antagonist at the human and mouse H_4_R in the luciferase reporter gene assay with comparable pK_B_ values of 7.81±0.19 and 7.58±0.13, respectively (Figure 5A, B, D). In contrast, at the rH_4_R JNJ 7777120 (**19**) acted as a partial agonist (α = 0.49±0.05) with a pEC_50_ value of 8.21±0.10 (Figure 5C). By analogy with ciproxifan, but much less pronounced, JNJ 7777120 (**19**) and thioperamide (**20**) produced receptor-independent increases in luciferase activity at concentrations ≥10 µM in control experiments using cells devoid of H_4_R expression (Figure 6). The corresponding values were therefore omitted in the construction of concentration-response curves of **19** and **20**, when studied in the antagonist mode (shown for JNJ 7777120 (**19**) in Figure 5D). Thioperamide (**20**) acted as an inverse agonist, achieving comparable pEC_50_ values at the human and mouse H_4_R (Figure 5A, B, Table 1), and revealed moderate antagonistic acitivity at the rH_4_R with a pK_B_ value of 6.89±0.14. The aminopyrimidine ST-1012 (**21**) acted as an inverse agonist at the hH_4_R, but revealed partial agonistic activity at the mouse and the rat H_4_R. The conformationally constrained aminopyrimidines A 943931 (**22**) and A 987306 (**23**) were inverse agonists at the hH_4_R and neutral antagonists at the rH_4_R.

## Discussion and Conclusions

### Assay Optimization

The pEC_50_ value of forskolin varied among the different transfectants probably due to different expression levels of the CRE-controlled luciferase. The concentration-response curve revealed a decline at forskolin concentrations higher than 10 µM. This decline of the forskolin effect became already obvious at concentrations >3.2 µM in the presence of 50 µM of the PDE inhibitor IBMX (data not shown), as already described for a CRE-directed luciferase reporter gene assay in Chinese hamster ovary cells (CHO) [37]. By analogy with a report by Kemp et al. [38] an activation of the inducable cAMP early repressor (ICER) may counteract the luciferase expression in HEK293T cells. Gα_i_-protein mediated inhibition of the cAMP synthesis as well as the signal-to-noise ratio was lowered by increasing concentrations of forskolin and IBMX. This was reflected by smaller relative effects and potencies of histamine (**1**) in the presence of increasing forskolin concentrations (Figure 3) and 50 µM of IBMX (Figure 4). Thus, high forskolin concentrations should be avoided and the altered potency of forskolin, when used in combination with IBMX, must be considered in this assay.

The co-expression of a CRE-controlled luciferase reporter gene with the human, mouse and rat H_4_R, respectively, in HEK293T cells enabled the functional analysis of H_4_R ligands. A set of 23 imidazole and non-imidazole ligands comprising agonists, inverse agonists and antagonists was investigated for ability to effect forskolin stimulated luciferase activity. The obtained pEC_50_ values or pK_B_ values were compared with ligand activities from different functional assay systems reported in literature.

### Off-target Effects

The luciferase stimulation becoming obvious at concentrations >1 µM of JNJ7777120 (**19**) and thioperamide (**20**) in cells expressing the H_4_R orthologs (cf. dashed lines in the concentration-response curves of **19** and **20** in Figure 5A-C) suggest inverse agonism. However, the investigation of selected compounds on HEK293T-CRE-Luc cells lacking the H_4_R (cf. Figure 6) revealed H_4_R-independent increase in luciferase activity. This effect was most prominent in case of ciproxifan (**12**), but also pronounced for **19** and **20**. Therefore, off-target effects should be taken into account to avoid misinterpretation of biological responses to such compounds at concentrations ≥10 µM.

### Activities at the Human H_4_ Receptor

**Figure 7:**
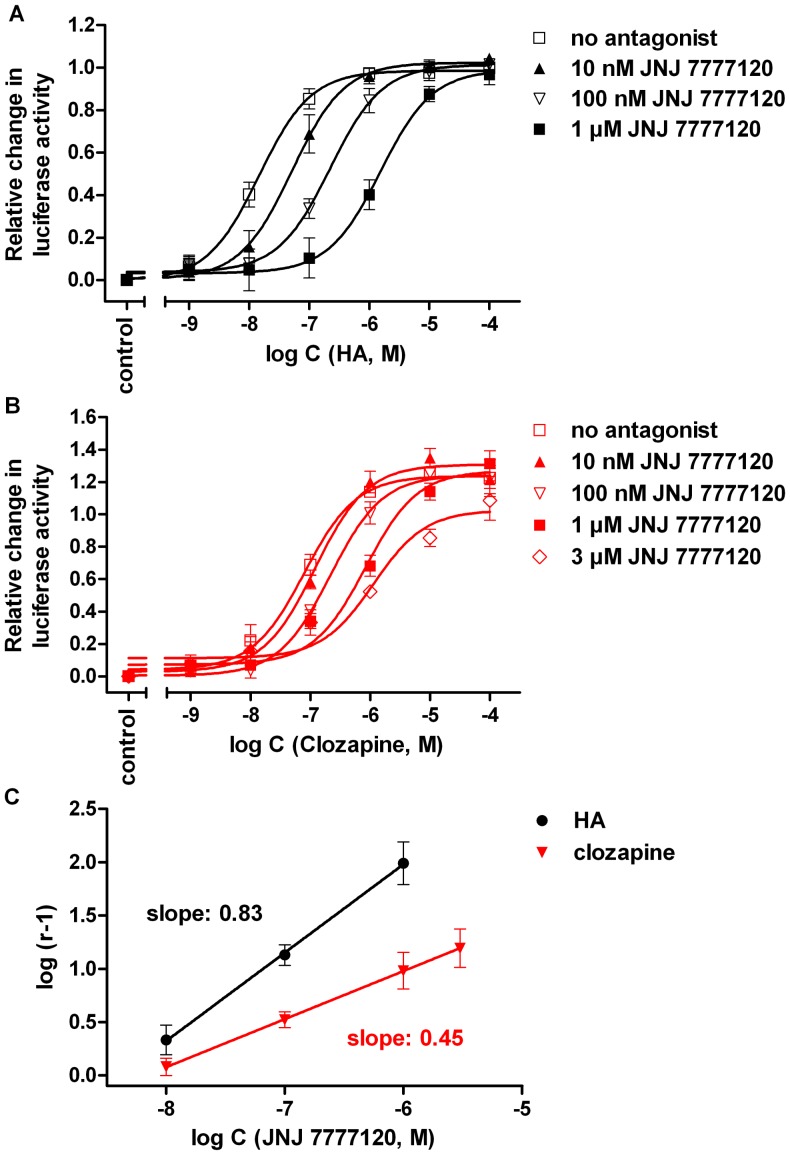
Inhibition of the response to histamine and clozapine by JNJ7777120. Concentration response curves of histamine (A) and clozapine (B) alone and in the presence of JNJ7777120 at increasing concentrations, determined on hH_4_R expressing HEK293T-CRE-Luc cells in the luciferase reporter gene assay, and corresponding Schild plots (C). The pA_2_ values determined for JNJ 7777120 from Schild regression were 8.39 (slope: 0.83±0.02) and 8.17 (slope: 0.45±0.01) versus histamine and clozapine, respectively. Data points shown are the mean ± SEM of at least three (histamine) or five (clozapine) independent experiments performed in triplicate.

Except for ST-1006 (**16**) [39] **,** all determined H_4_R ligand activities at the hH_4_R were in agreement with results reported in literature [20], [23], [39]–[42]. However, a tendency toward elevated intrinsic activities was observed. Contrary to partial agonistic activity of immepip (**6**) and clobenpropit (**9**) in the [^35^S]GTPγS binding assay on membrane preparations of H_4_R expressing Sf9 cells (α = 0.81and 0.45, respectively) (Table 2), full agonism at the hH_4_R was determined in the luciferase assay. Iodophenpropit (**10**), described as a neutral antagonist [40], exerted strong partial agonistic activity at the hH_4_R in the present study. Partial agonistic activity was also determined for iodophenpropit (**10**) in a Ca^2+^ mobilization assay in HEK293 cells, co-transfected with the hH_4_R and the chimeric G-protein G_qi5_ [5]. ST-1006 (**16**) had low intrinsic activity in the [^32^P]GTPase and [^35^S]GTPγS binding assay at the hH_4_R [39], but was an almost full agonist in the luciferase assay. The increased intrinsic activity was accompanied with a decrease in potency of about one order of magnitude. In case of clozapine (**17**), the maximal agonistic response surpassed that of histamine by 30%. In control experiments on HEK293T-CRE-Luc cells devoid of the H_4_R, clozapine (**17**) at concentrations as high as 100 µM caused an increase in CRE-activity by up to 17% (data not shown). The effect of clozapine on hH_4_R expressing cells was antagonized by JNJ 7777120 in a concentration-dependent manner, indicating that the (super)agonistic effect was receptor mediated (Figure 7). Using histamine or clozapine as H_4_R agonists revealed approximately the same pA_2_ value for JNJ 7777120 (pA_2_ values: 8.39 and 8.17). However, compared to the concentration response curve of histamine in the presence of JNJ 7777120 (Figure 7A), the extent of rightward shift was smaller in case of clozapine (Figure 7B), resulting in different slopes (0.83 compared to 0.45) of the corresponding Schild plots (Figure 7C). This may be taken as a hint that histamine and clozapine activate the H_4_R not exactly in the same way. However, due to the pleiotropic character of clozapine (**17**), effects mediated by targets other than the H_4_R must be taken into account. Most probably, increased intrinsic activities in the luciferase assay compared to more proximal readouts are caused by amplifications in signaling downstream from G-protein activation [30], [37]. For instance, in functional assays on Sf9 cell membranes, ST-1006 (**16**) [39] and clozapine (**17**) [43] showed only partial agonism (Table 2).

The constitutive activity of the hH_4_R, obvious from inverse agonism of thioperamide (**20**), was rather low compared to functional assays on Sf9 cell membranes [23], [34]. In accordance with reported data ST-1012 (**21**) acted as an inverse hH_4_R agonist in the [^35^S]GTPγS assay [39], and JNJ 7777120 (**19**) behaved as a neutral hH_4_R antagonist [18], [40]. Inverse agonism was also found for A 943931 (**22**) and A 987306 (**23**) in the luciferase (Table 1) and the GTPγS assay (Table 2), whereas neutral antagonism was observed in Ca^2+^ (FLIPR) assays [44], [45].

### Activities at Rodent H_4_ Receptors

Comparing the results from the luciferase assay on mouse and rat H_4_R with data from other functional assays revealed marked differences. The potencies of histamine (**1**), 5(4)-methylhistamine (**5**), immepip (**6**), UR-PI294 (**13**), VUF 8430 (**15**) and clozapine (**17**) were significantly higher compared to the [^32^P]GTPase [23] and [^35^S]GTPγS binding assay (Table 2). By contrast, the agonist potencies of histamine (**1**), (R)-α-methylhistamine (**2**), N^α^-methylhistamine (**4**) and imetit (**8**) were consistent or lower compared to results from a Ca^2+^ assay using HEK293 cells, co-expressing the mouse or the rat H_4_R with Gα_qi5_ [2], [46]. For example, in the luciferase assay the pEC_50_ values of histamine (**1**) were in good agreement with results from the Ca^2+^ assay at the mouse and rat H_4_R (7.23 and 6.49, respectively) [46], but distinctly higher compared to pEC_50_ values from the [^32^P]GTPase assay (5.81 and 5.23, respectively) [23]. UR-PI294 (**13**) achieved pEC_50_ values >8 at the hH_4_R, mH_4_R and rH_4_R in the luciferase assay, whereas the [^32^P]GTPase assay revealed dramatic differences in pEC_50_ values (8.52, 6.50 and 4.64, respectively) [23]. The potency of imetit (**8**) was lower compared to the Ca^2+^ assay in HEK293 cells (pEC_50_ values: 7.4 and 7.2 vs. 8.1 at both receptors) [20]. Whereas being full agonists in the luciferase assay, (R)-α-methylhistamine (**2**), N^α^-methylhistamine (**4**) and imetit (**8**) only reached 75–80% of the maximal Ca^2+^ response at the mH_4_R and 30–50% at the rH_4_R [20].

The pK_B_ values of neutral antagonists, such as iodophenpropit (**10**) at the mouse and rat H_4_R as well as thioperamide (**20**) and UR-PI376 (**14**) at the rH_4_R were comparable to those determined in the [^35^S]GTPγS binding assay (Table 2). Mouse and rat H_4_R-mediated inhibition of forskolin-stimulated luciferase activity in HEK293T-CRE-Luc cells resulted in higher potencies compared to functional assays using Gα-protein activation as readout. This suggests that signal amplification or concomitant activation of different signaling pathways potentiates the inhibition of the luciferase activity. For example, the cAMP pathway may be modulated by a cross-talk with Ca^2+^ signaling elicited by activation of phospholipase C (PLC) [47]. Ca^2+^ is an inhibitor of (forskolin) stimulated and Ca^2+^ sensitive adenylate cyclases type V/VI [48]–[50], which are endogenously expressed in HEK293T cells [51] and interact with the Gα_i_ protein [52]. Furthermore, the relevance of this crosstalk with regard to the cAMP signaling pathway of G-protein coupled receptors (GPCRs) was demonstrated by the inhibitory effect of the activated Gα_q_ coupled histamine H_1_R on the cAMP level in U373 MG cells [53] and, more importantly, by a crosstalk between the Gα_i_ coupled M_2_ mACh receptor and the Gα_q_ coupled M_3_ mACh receptor. In the latter case the inhibition of forskolin-stimulated cyclic AMP accumulation was facilitated at low agonist concentrations [54]. Further studies on the influence of Ca^2+^ are needed to clarify, whether only the rodent H_4_Rs are concerned, since agonist potencies at the hH_4_R were consistent with data from the [^32^P]GTPase and [^35^S]GTPγS binding assay (Table 2). Very recently, investigations on human esosinophils revealed a lower Ca^2+^ response to stimulation by histamine (**1**) and UR-PI376 (**14**) compared to the chemokine eotaxin via the CCR3 receptor [55]. This may be interpreted as a hint to minor contribution of Ca^2+^ signaling to the overall H_4_R mediated response, at least in native human cells. The presence of a range of alternative signaling pathways for the H_4_R in living cells was underlined recently by the Gα independent ß-arrestin recruitment of several H_4_R ligands [26], [27].

The results for the standard antagonist JNJ 7777120 (**19**) at the mouse and rat H_4_R compared with data reported for other functional assays revealed discrepancies, too. In the luciferase assay JNJ 7777120 (**19**) acted as a neutral antagonist at the mH_4_R, but as a potent partial agonist at the rH_4_R. Antagonistic activity at both receptors was found in a CRE-driven β-galactosidase assay in SK-N-MC cells [18] and in a Ca^2+^ assay in HEK293 cells [46], whereas partial agonistic activity was determined at the mouse and rat receptor in the [^32^P]GTPase [23] and [^35^S]GTPγS binding assay (Table 2). The pK_B_ value at the mH_4_R in the luciferase assay is consistent with the pK_B_ value in the Ca^2+^ assay [46], whereas the agonistic potency at the rH_4_R is about two orders of magnitude higher compared to the [^32^P]GTPase assay [23]. Discrepancies between the H_4_R orthologs in the different assay systems may result from differential equilibria between the active and inactive states of the H_4_R in the different assay systems as described recently [56]. In the luciferase assay, the constitutive activity, reflected by the inverse agonism of compounds **20–23**, was considerably higher for the mH_4_R than for the rH_4_R. At the latter JNJ 7777120 shifted the equilibrium toward the active state, becoming obvious as agonistic activity. Inversely, ST1006 (**16**), a potent agonist a human and mouse H_4_R, showed considerable inverse agonism at the rH_4_R. Thus, the outcome of studies in translational animal models cannot be unequivocally predicted by in vitro experiments, but such data may help to interprete conflicting results such as the pro-inflammatory effect of JNJ 7777120 (**19**) in a rat conjunctivitis model [17].

**Figure 8:**
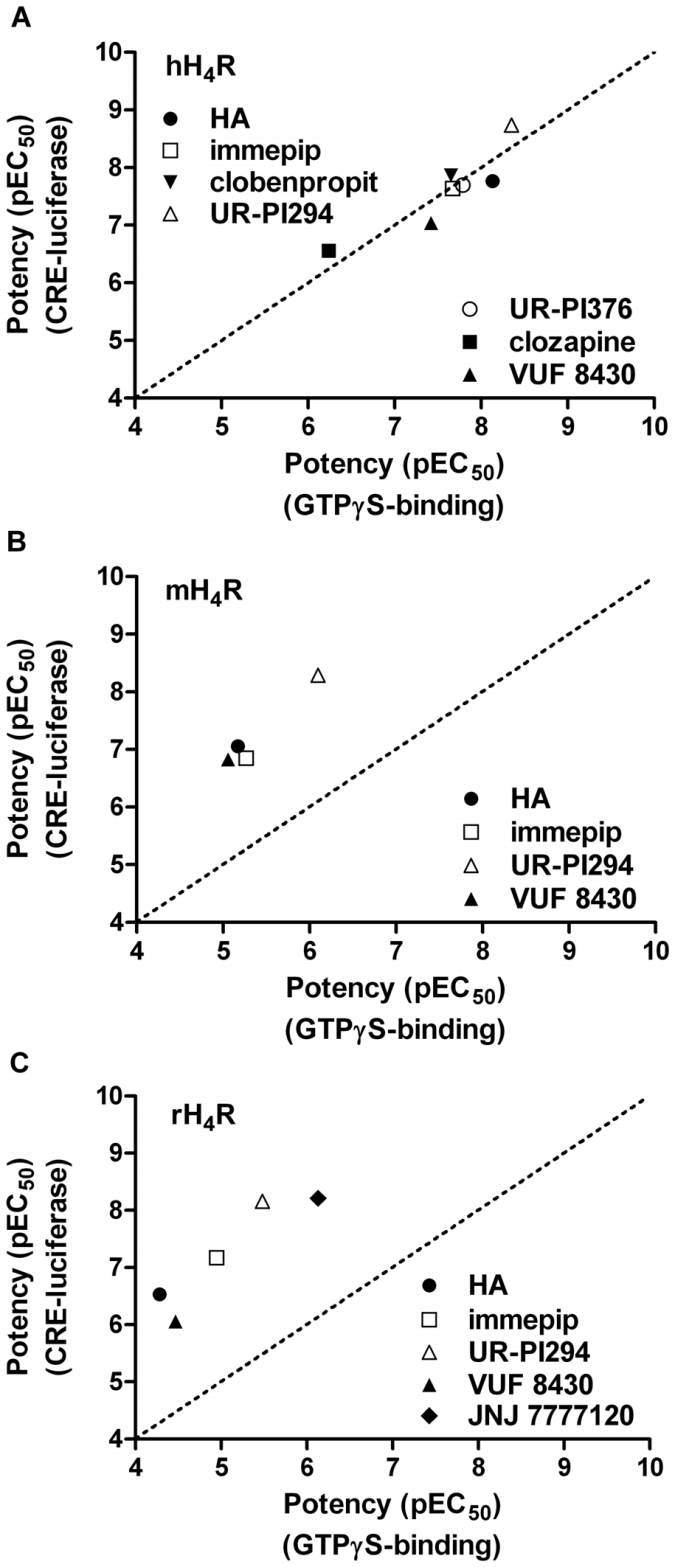
Comparison of distal and proximal readouts. Correlation between agonist potencies in the luciferase reporter gene assay and the [^35^S]GTPγS assay at the (A) hH_4_R (slope: 0.90±0.20; r^2^ = 0.80), (B) mH_4_R (slope: 1.431±0.23; r^2^ = 0.95) and (C) rH_4_R (slope: 1.171±0.28, r^2^ = 0.85).

In case of agonism at the human H_4_R, the data correlate very well with data provided by more proximal readouts such as GTPase activity or GTPγS binding (Table 1, Table 2, Figure 8A). This also holds for the rank order of agonists at the mouse and rat H_4_R (Table 1, Table 2, Figure 8B,C), however, the potencies are up to 100-fold higher in the luciferase assay.

## Conclusions

The reporter gene (luciferase) assay in HEK293T cells allows for the quantification of agonistic, inverse agonistic and antagonistic activity at the H_4_R species orthologs in a highly sensitive and reliable manner. In view of significantly increased potencies and efficacies of agonists, especially at the rodent H_4_R orthologs, obviously, there is a positive effect on the readout by activation/amplification of or cross-talk between different signaling pathways in the luciferase reporter gene assay compared to more proximal functional assays on Sf9 cell membranes. It has now become clear that unequivocal characterization of H_4_R ligands as agonists, antagonists or inverse agonists in assays using a single readout is impossible. Thus, ligands have to be examined in multiple assays. But at present, it seems impossible to predict the value of the in vitro data with respect to translational animal studies and their clinical relevance.
